# Recent Progress of Biomaterial-Based Hydrogels for Wearable and Implantable Bioelectronics

**DOI:** 10.3390/gels11060442

**Published:** 2025-06-09

**Authors:** Baojin Chen, Yan Zhu, Renjie Yu, Yunxiang Feng, Zhenpeng Han, Chang Liu, Pengcheng Zhu, Lijun Lu, Yanchao Mao

**Affiliations:** Key Laboratory of Materials Physics of Ministry of Education, School of Physics, Zhengzhou University, Zhengzhou 450001, China

**Keywords:** biomaterials, hydrogel, wearable, implantable, bioelectronics

## Abstract

Bioelectronics for wearable and implantable biomedical devices has attracted significant attention due to its potential for continuous health monitoring, early disease diagnosis, and real-time therapeutic interventions. Among the various materials explored for bioelectronic applications, hydrogels derived from natural biopolymers have emerged as highly promising candidates, owing to their inherent biocompatibility, mechanical compliance akin to biological tissues, and tunable structural properties. This review provides a comprehensive overview of recent advancements in the design and application of protein-based hydrogels, including gelatin, collagen, silk fibroin, and gluten, as well as carbohydrate-based hydrogels such as chitosan, cellulose, alginate, and starch. Particular emphasis is placed on elucidating their intrinsic material characteristics, modification strategies to improve electrical and mechanical performance, and their applicability for bioelectronic interfaces. The review further explores their diverse applications in physiological and biochemical signal sensing, bioelectric signal recording, and electrical stimulation. Finally, current challenges and future perspectives are discussed to guide the ongoing innovation of hydrogel-based systems for next-generation bioelectronic technologies.

## 1. Introduction

The convergence of artificial intelligence, human–machine interfaces (HMIs), and next-generation healthcare technologies is reshaping the landscape of biomedical innovation [1,2,3,4]. This progression is driving the demand for personalized medicine and continuous health monitoring, necessitating the development of intelligent devices capable of reliable operation in dynamic biological environments. A diverse array of wearable and implantable health monitoring devices has been developed to meet this demand, including respiratory, electrophysiological, glucose, and motion monitors. These devices have enabled real-time tracking of vital physiological signals, offering new opportunities for early diagnosis and preventive healthcare [5,6]. However, many existing systems are hindered by their rigid architectures, limited stretchability, and insufficient long-term biocompatibility, compromising their performance under continuous mechanical deformation and hindering seamless integration with soft biological tissues [7,8,9]. These challenges highlight the pressing need for advanced materials that can conform intimately to the body while maintaining functional stability. As bioelectronic interfaces continue to evolve, the integration of soft, bioinspired materials into electronic systems has emerged as a pivotal strategy for advancing next-generation biomedical technologies [10,11,12].

In response to these material challenges and integration demands, bioelectronics has emerged as a transformative interdisciplinary platform, integrating advances from material science, electronics, and biomedicine [13,14,15,16]. However, a key challenge is that interface materials seamlessly integrate with biological tissues without eliciting adverse responses. Although conventional electronic materials often exhibit excellent electrical conductivity, their mechanical mismatch with soft tissues frequently undermines performance and limits long-term biocompatibility [17,18,19]. Hydrogels derived from biomaterials have shown significant promise in addressing these limitations. Their soft mechanical properties facilitate stress-free interfacing with tissues, while their intrinsic ionic conductivity supports efficient transduction of physiological signals [20,21]. Natural biomaterial-based hydrogels, particularly those derived from proteins and polysaccharides, offer bioactive moieties and versatile functional groups [22,23,24,25]. These characteristics enable targeted tuning of electrical, mechanical, and biological properties through chemical crosslinking, conductive network embedding, or topological structuring [26,27,28,29,30].

**Figure 1:**
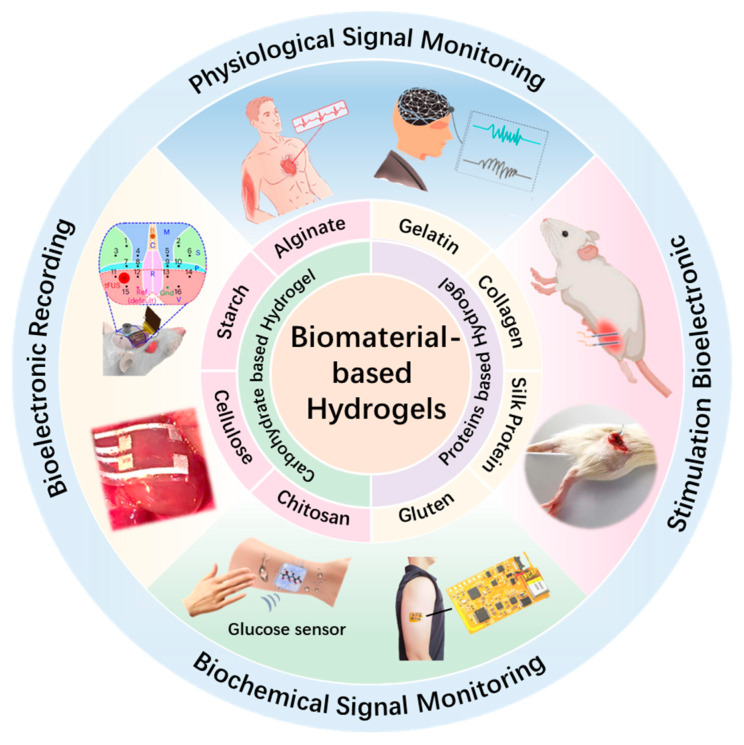
Recent progress of biomaterial-based hydrogels for wearable and implantable bioelectronics. Physiological Signal Monitoring: reproduced with permission [31]. Copyright 2023, Elsevier. Reproduced with permission [32]. Copyright 2023, Wiley. Stimulation Bioelectronic: reproduced with permission [33]. Copyright 2023, Springer Nature. Reproduced with permission [34]. Copyright 2025, Elsevier. Biochemical Signal Monitoring: reproduced with permission [35]. Copyright 2023, Cell Press. Reproduced with permission [36]. Copyright 2023, Elsevier. Bioelectronic Recording: reproduced with permission [37]. Copyright 2023, Springer Nature. Reproduced with permission [15]. Copyright 2024, Springer Nature.

This review provides a comprehensive summary of recent advances in hydrogel materials for next-generation bioelectronic devices. As shown in Figure 1, the discussion focuses on two primary categories of natural biopolymers: polysaccharides such as cellulose, chitosan, alginate, and starch, and proteins such as gelatin, collagen, silk fibroin, and gluten. Their intrinsic properties, including biocompatibility, tissue-matching mechanical compliance, and tunable structure, are carefully discussed. On this basis, biomaterial-based hydrogels are analyzed for applications in electrophysiological and biochemical signal monitoring, bioelectric stimulation, and bioelectric recording. Furthermore, key challenges, such as long-term stability, material performance degradation, and seamless integration with electronic components, are discussed. Finally, future perspectives are proposed to guide the ongoing development of multifunctional hydrogel-based platforms for next-generation bioelectronics.

## 2. Biomaterial-Based Hydrogel

Biomaterial-based hydrogels, an emerging class of intelligent soft materials, offer a promising platform for integrating biofunctionality with tunable physicochemical properties through molecular engineering [38,39,40]. These three-dimensional (3D) networks, composed of natural macromolecules such as proteins and polysaccharides, are typically stabilized via crosslinking strategies, including hydrogen bonding, electrostatic interactions, and covalent linkages [41,42,43]. In representative hydrogel systems, common synthesis routes mainly include chemical crosslinking, physical crosslinking, and enzymatic reactions. Chemical crosslinking, for example, is often achieved by aldehyde–amine reaction, free radical polymerization, or isocyanate reaction to achieve the connection between polymer chains. The crosslinking conditions usually involve the molar ratio of reactants, solution pH, reaction temperature, and reaction time, which directly affect the mechanical properties, gelation rate, and microstructure of hydrogels [17,44]. This molecular design strategy ensures intrinsic biocompatibility while enabling precise tuning of performance parameters such as conductivity and Young’s modulus through the modulation of polymer architecture and the incorporation of functional groups [45,46]. Compared to conventional rigid electronic materials such as silicon or metallic components, hydrogels exhibit superior mechanical compatibility with soft biological tissues [47,48,49].

In implantable bioelectronics, the dynamic viscoelasticity of hydrogels alleviates mechanical mismatch at tissue interfaces, enabling over 80% reduction in interfacial stress and strain through optimized energy dissipation mechanisms [15,50]. Their inherent stretchability and viscoelasticity further support their application in high-fidelity physiological signal monitoring. By integrating conductive nanomaterials, dynamic covalent linkages, and tissue-interactive functional groups, hydrogel-based bioelectronic systems have been engineered with multimodal sensing capabilities and seamless integration with biological tissues [26,51,52].

Despite these advantages, current biomaterial-based hydrogel synthesis strategies still face significant challenges in terms of time efficiency and scalability. Traditional methods such as physical crosslinking, chemical crosslinking, and self-assembly can effectively tailor hydrogel properties on a laboratory scale, but often suffer from long synthesis times, complex reaction conditions, and limited batch-to-batch reproducibility. In addition, achieving consistent performance in large-scale mass production remains a persistent obstacle. However, striking a balance between performance reliability, cost efficiency, and environmental sustainability remains a central challenge in driving hydrogel-based technologies towards practical bioelectronic applications.

### 2.1. Protein-Based Hydrogels

Hydrogels derived from proteins such as collagen, gelatin, and silk fibroin have drawn considerable attention for use in implantable and wearable bioelectronic devices, owing to their intrinsic biological activity and customizable structural properties [53,54,55]. At the molecular level, protein chains are capable of self-assembly or chemical crosslinking to form 3D networks, in which diverse amino acid sequences create biomimetic microenvironments that resemble native tissues [56]. Furthermore, functionalization strategies, such as the integration of conductive nanowires, conjugated polymers, and ionic groups, have been employed to precisely modulate the electrical conductivity of protein-based hydrogels [54]. Additionally, these hydrogels exhibit mechanical compatibility with soft biological tissues [57,58,59,60], making them excellent candidates for next-generation wearable and implantable electronics.

#### 2.1.1. Gelatin

Gelatin, a biopolymer naturally produced through the controlled hydrolysis of collagen, retains the characteristic (Gly-X-Y)n triplet sequence in its molecular chains [61,62]. This characteristic sequence imparts excellent biocompatibility and biomimetic features to gelatin, making it highly suitable as a foundational material for fabricating functional hydrogels. Consequently, gelatin has been widely utilized as a structural platform in the design and development of advanced hydrogel systems [22,63]. In particular, the pore size distribution and Young’s modulus of gelatin-based 3D networks have been finely tuned through the synergistic control of physical crosslinking and chemical modification. These tunable physicochemical characteristics enable dynamic modulation of hydrogel properties, establishing gelatin-based hydrogels as promising candidates for flexible bioelectronic devices.

**Figure 2:**
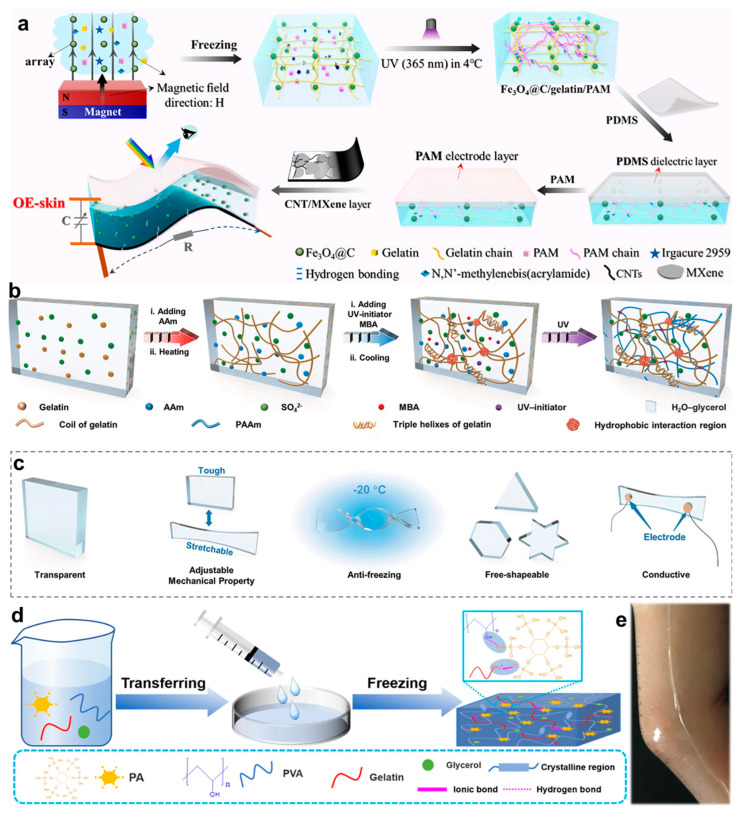
Fabrication procedures and functional demonstrations of gelatin-based hydrogels. (**a**) Schematic illustration of the fabrication process for the multilayered chromotropic OE-skin [65]. (**b**) Synthetic route of PGAOH. (**c**) Functional demonstration matrix showcasing the multi-responsive properties of PGAOH [63]. (**d**) Schematic representation of the preparation process of PPG hydrogel. (**e**) Photographs of PPG hydrogel attached to human joints [66]. All pictures have been adopted with permission.

However, the intrinsic electrical insulation of native gelatin limits its direct application in conductive bioelectronics. To overcome this limitation, conductive nanomaterials such as MXenes and polypyrrole nanowires have been incorporated to construct biomimetic ionic–electronic dual conduction pathways [26,64]. Subsequently, several representative studies are summarized to demonstrate recent progress in gelatin-based hydrogels for multifunctional bioelectronic applications. Zhang et al. [65] developed a multimodal responsive optical/electrical skin (OE-skin) through a bio-inspired hierarchical design strategy (Figure 2a). The OE-skin comprises a three-layered architecture: The top layer integrates an ionic electrode with an elastic dielectric interface for pressure sensing. The middle layer incorporates a magnetic photonic crystal in a dual-network hydrogel for dynamic color switching. The bottom layer features a carbon nanotube/MXene-based conductive film with microcrack structures for strain sensitivity. This gradient-modulus configuration optimizes mechanical adaptability, presenting a new paradigm for cross-scale signal coupling in intelligent robotics (Figure 2a). In addition, Yu et al. [63] synthesized a poly(acrylamide)/gelatin/ammonium sulfate organohydrogel (PGAOH) using a facile one-step fabrication strategy (Figure 2b). In this system, acrylamide (AM) improves gelatin dispersibility in high-salt environments, which significantly reduces gelation time, allows for precise volume control, and promotes the formation of a robust double-network structure that enhances mechanical properties. As shown in Figure 2c, the resulting PGAOH exhibits multiple functional characteristics, including anti-freezing behavior, excellent stability, ionic conductivity, sensitivity, moldability, transparency, and elasticity. These properties have been validated in wireless virtual reality gaming applications. Furthermore, Liu et al. [66] developed a thermoresponsive poly(vinyl alcohol)/phytic acid/gelatin (PPG) composite hydrogel based on dynamic hydrogen bonding (Figure 2d). The multivalent hydrogen bond network enables reversible phase transitions within the physiological temperature range (25–37 °C), enabling controllable adhesion behavior. As shown in Figure 2e, PPG hydrogel sensors adhered to finger joints demonstrate stable monitoring of flexion–extension motions over 12 h, indicating substantial potential for sustainable bioelectronic systems.

#### 2.1.2. Collagen

Collagen, a natural structural protein, possesses a unique triple-helical molecular conformation that imparts distinct biofunctional properties and physiological compatibility [67,68]. In bioelectronic applications, collagen-based hydrogels have garnered significant interest, owing to their adjustable network configurations and exceptional biological properties [69,70]. These hydrogels are typically constructed through the synergistic combination of physical self-assembly and chemical crosslinking, enabling precise regulation of mechanical and electrical properties.

**Figure 3:**
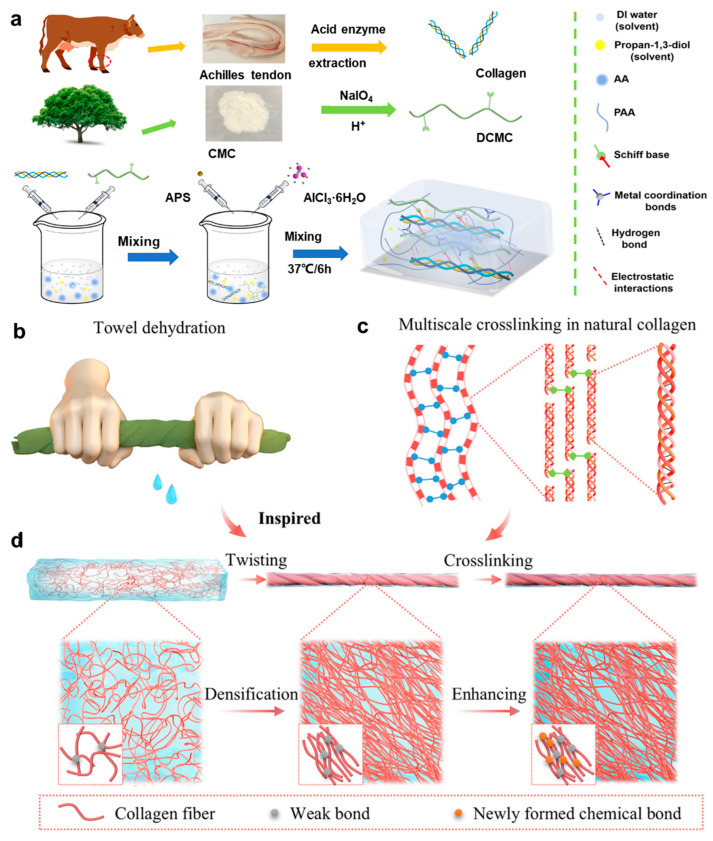
Structural design and functional demonstration of collagen-based hydrogels. (**a**) Schematic illustration of the fabrication process for CDPAP hydrogels [23]. (**b**) Photographs showing towel-inspired mechanical manipulation, including twisting and squeezing deformations. (**c**) Hierarchical multiscale crosslinking network in natural collagen fibrils. (**d**) Bioinspired processing strategy for collagen hydrogels [72]. All pictures have been adopted with permission.

However, conventional collagen-based hydrogels often suffer from mechanical limitations, restricting their long-term stability under dynamic physiological conditions. To address these challenges, conductive polymers and ionic liquids have been incorporated into collagen matrices, resulting in double-network hydrogel architectures [68,71]. This section highlights recent advances in strategies for enhancing the mechanical performance of collagen-based hydrogels. Ling et al. [23] designed a 3D interpenetrating collagen-based self-healing conductive organohydrogel (CDPAP) by integrating collagen with acrylic acid, dialdehyde carboxymethyl cellulose, and Al^3+^ ions in a water/propylene glycol cosolvent (Figure 3a). This system, which leverages a dual crosslinking mechanism based on dynamic imine bonds and metal coordination, exhibits high tensile strength, rapid self-healing capability, and robustness under extreme environmental conditions. Compared with conventional collagen hydrogels, the CDPAP hydrogel demonstrates superior mechanical resilience and operational stability. In addition, Li et al. [72] introduced a novel biomimetic strategy inspired by the “towel-twisting” dehydration mechanism to achieve controlled fiber alignment. A two-step process combining twist-induced fiber orientation with covalent network reinforcement was established (Figure 3b–d). This method allows for accurate regulation of fiber orientation and facilitates swift covalent crosslinking, which significantly enhances both mechanical strength and toughness while maintaining excellent biocompatibility. Aligned collagen networks fabricated via this strategy exhibited mechanical properties several times greater than those of randomly oriented structures.

#### 2.1.3. Silk Fibroin

Silk fibroin has emerged as a promising natural material for bioelectronic applications, forming a 3D *β*-sheet crystalline network through the self-assembly of its heavy- and light-chain polypeptides, which provides structural stability and tenability [24,54,73]. The high glycine (~45%) and alanine (~30%) content in silkworm silk contributes to molecular flexibility, thereby facilitating *β*-sheet formation. Consequently, silk fibroin exhibits favorable mechanical strength and can be functionally tuned through chemical modification, physical processing, or molecular engineering strategies [51,55,74]. These hydrogels combine biocompatibility, breathability, and biodegradability, making them ideal as flexible substrates for next-generation implantable electrodes and intelligent wearable sensors.

**Figure 4:**
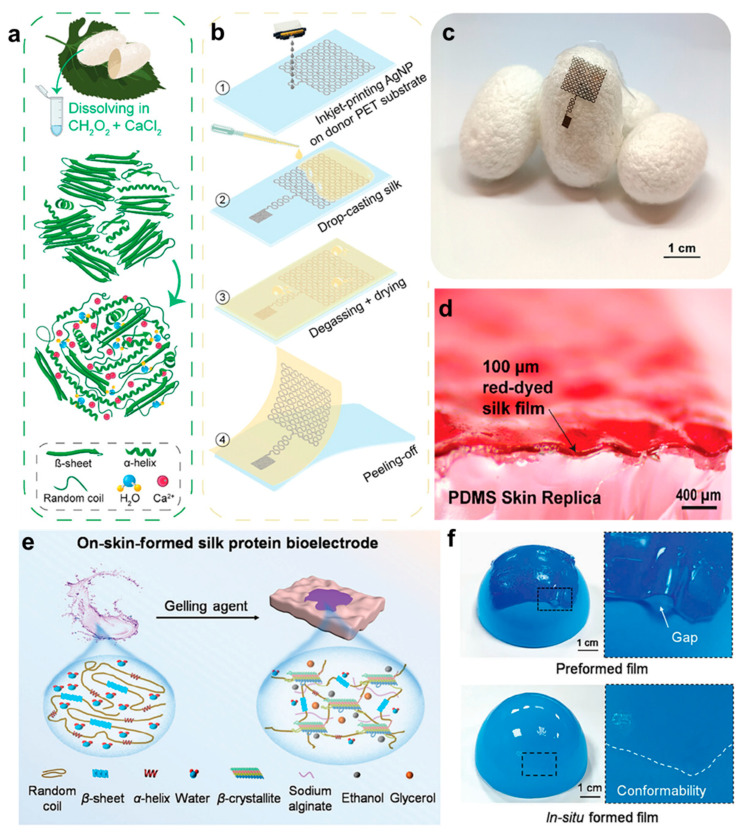
Fabrication principles and morphological characteristics of silk protein-based hydrogels. (**a**) Schematic illustration of Ca^2+^-induced structural transition in silk proteins. (**b**) Micro-/nanopatterning technique employed in the fabrication of silk-based bioelectronic devices. (**c**) Optical image demonstrating the direct integration of silk bioelectronics. (**d**) Cross-sectional SEM image between red-dyed silk and PDMS [75]. (**e**) Schematic illustration of the synthesis route of the on-skin-formed silk protein (OSF-SP) bioelectrode and its secondary structure transition in the gelation process. (**f**) Photographs of a preformed film and an in situ-formed film covering the surface of a ball [55]. All pictures have been adopted with permission.

Recent research has highlighted the versatility of silk fibroin in constructing advanced bioelectronic systems. For example, Mirbakht et al. [75] developed a silk fibroin-based electronic skin by modulating its *β*-sheet crystallization behavior (Figure 4a). Inkjet printing enabled in situ patterning of silk fibroin into micron-scale circuits, enhancing fabrication efficiency and increasing integration density (Figure 4b). At the material level, the system exhibited superior self-adhesiveness, breathability, and stretchability compared with conventional substrates, closely mimicking the mechanical behavior of human skin. At the system level, high-resolution circuits fabricated by inkjet printing are shown in Figure 4c, while robust bonding between the circuit and the flexible substrate, critical for stable biosignal monitoring, is illustrated in Figure 4d. These features enabled reliable, noninvasive monitoring even on challenging skin regions, such as hairy or perspiration-prone areas. Yang et al. [55] report a silk protein-based bioelectrode that forms directly on the skin, offering a seamless and conformable interface for improved electrophysiological signal acquisition (Figure 4e). Unlike traditional electrodes, this system adapts well to hairy and irregular skin surfaces, significantly enhancing signal quality with a signal-to-noise ratio (SNR) improvement reaching 38.9%. The fluid–gel transition, triggered by ethanol and sodium alginate, ensures robust skin contact (Figure 4f). In a related study, Wang et al. [54] developed a programmable silk fibroin system exhibiting electrical responsiveness and structural reconfigurability. This was achieved via isoelectric point migration driven by low-voltage-induced pH gradients, enabling precise control over the 3D structure. The in situ programming strategy enabled reversible, multimodal deformation in three dimensions, allowing real-time structural reconfiguration in response to electrical stimuli. Therefore, dynamic mechanical matching with biological tissues under diverse application scenarios was realized.

#### 2.1.4. Gluten

**Figure 5:**
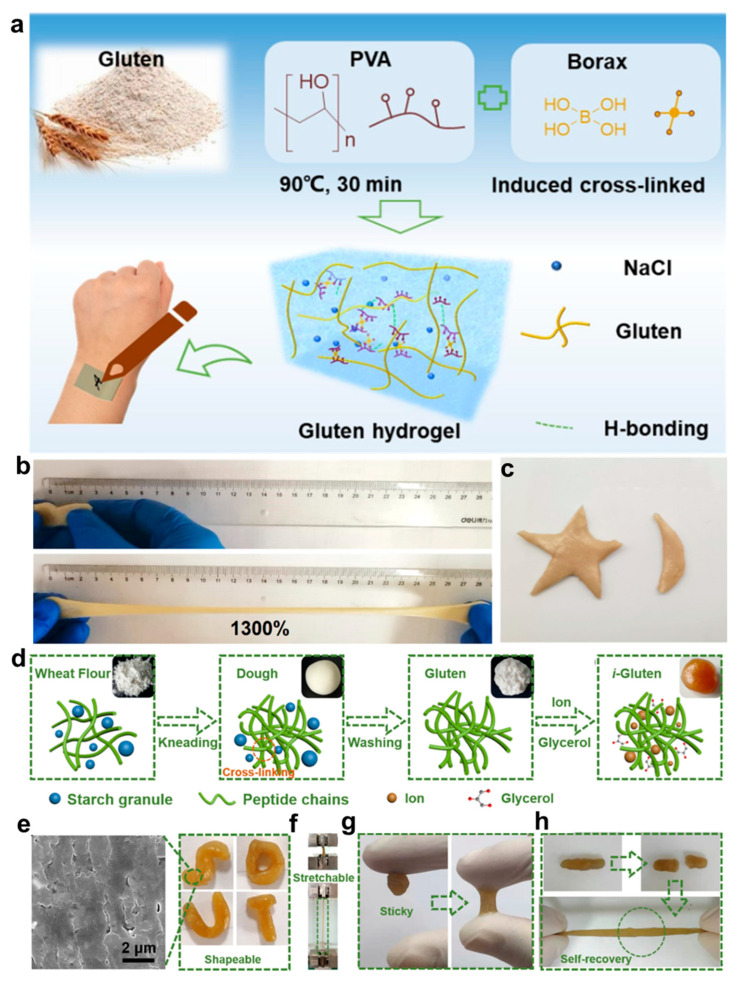
Fabrication strategy and multifunctional performance of glutenin-based hydrogels. (**a**) Schematic illustration of the preparation procedure of the gluten-based e-skin. (**b**) Photographs of stretched gluten hydrogel. (**c**) Different shapes of gluten hydrogel photographs [79]. (**d**) Schematic illustration of the fabrication process of conductive *i*-Gluten hydrogels. (**e**) Optical and SEM images displaying the morphology of *i*-Gluten. (**f**) Photographs demonstrating the excellent stretchability of *i*-Gluten. (**g**) Photographs showing the adhesive capability of *i*-Gluten on various substrates. (**h**) Photographs highlighting the self-recovery performance of *i*-Gluten after mechanical damage [80]. All pictures have been adopted with permission.

Gluten is a protein complex naturally extracted from cereals such as wheat and barley, and is abundant in functional groups such as amino and carboxyl moieties. These chains can form 3D dynamic networks via physical or chemical crosslinking, thereby endowing hydrogel systems with tunable mechanical properties [76,77,78]. In flexible bioelectronics, gluten-based hydrogels are considered promising materials for wearable sensors and bio-integrated electrodes due to their tissue-like flexibility, strong interfacial adhesion, and stable ionic conductivity. Chen et al. [79] developed a double-network hydrogel composed of polyvinyl alcohol and gluten protein, crosslinked with borax (Figure 5a). The hydrogel demonstrates exceptional mechanical performance, with stretchability up to 1300% and excellent shape moldability (Figure 5b,c). These properties are largely attributed to the incorporation of NaCl, which enhances polymer chain interactions and network dynamics. Additionally, the electronic skin exhibits both biodegradability and biocompatibility, positioning it as a strong candidate for environmentally sustainable wearable electronic applications. Meanwhile, Han et al. [80] developed an ionically conductive gluten (*i*-Gluten) hydrogel by leveraging synergistic coordination between glycerol and ionic species, enabling dynamic reconfiguration of the peptide network (Figure 5d). This strategy imparted self-healing capability and robust interfacial adhesion to the hydrogel (Figure 5e–h). Due to its mechanical compatibility with skin, the material exhibited stable performance across a wide range of physiological activities, from gross limb movements to fine EMG signals, and maintained functionality over 3000 mechanical cycles.

### 2.2. Polysaccharide-Based Hydrogels

Polysaccharide-based hydrogels, such as those derived from chitosan (CS), hyaluronic acid, and alginate (Alg), have attracted growing interest in the field of wearable bioelectronics, due to their natural abundance, biodegradability, and structurally diverse molecular frameworks [41,81,82]. Although polysaccharide-based materials are often considered to be abundantly sourced and eco-friendly, their cost is not absolutely inexpensive, but rather has some economic advantages over other biomedical polymers or functional synthetic polymers. Enriched with reactive functional groups such as hydroxyl and amino moieties, these hydrogels provide a versatile platform for chemical modification. By grafting conductive monomers or incorporating carbon-based nanomaterials and metal nanoparticles, composite systems with simultaneous ionic and electronic conductivity can be readily engineered [34,83]. Despite these advantages, achieving sufficient mechanical durability and multifunctionality suitable for dynamic biological environments remains challenging. Rational structural design strategies, such as utilizing hydrogen bonding, ionic crosslinking, or dynamic covalent bonds, can markedly enhance their stretchability and self-healing capabilities [82,84,85]. These mechanical properties are particularly desirable for accommodating complex deformations associated with joint movements and epidermal dynamics. Compared with conventional elastomers, polysaccharide hydrogels exhibit superior adaptability under repetitive strain, facilitating their integration into soft bioelectronic interfaces [86,87,88]. In summary, these characteristics make polysaccharide hydrogels promising candidates for the development of next-generation wearable and implantable bioelectronic devices.

#### 2.2.1. Chitosan

Chitosan is an alkaline polysaccharide of natural origin, containing abundant amino and hydroxyl groups along its molecular chain [88,89,90]. These functional groups enable the formation of a 3D network structure through physical or chemical crosslinking. This structure not only imparts excellent water absorption capacity and mechanical extensibility but also significantly improves ionic conductivity when integrated with conductive components such as polyaniline or carbon nanotubes [91,92].

**Figure 6:**
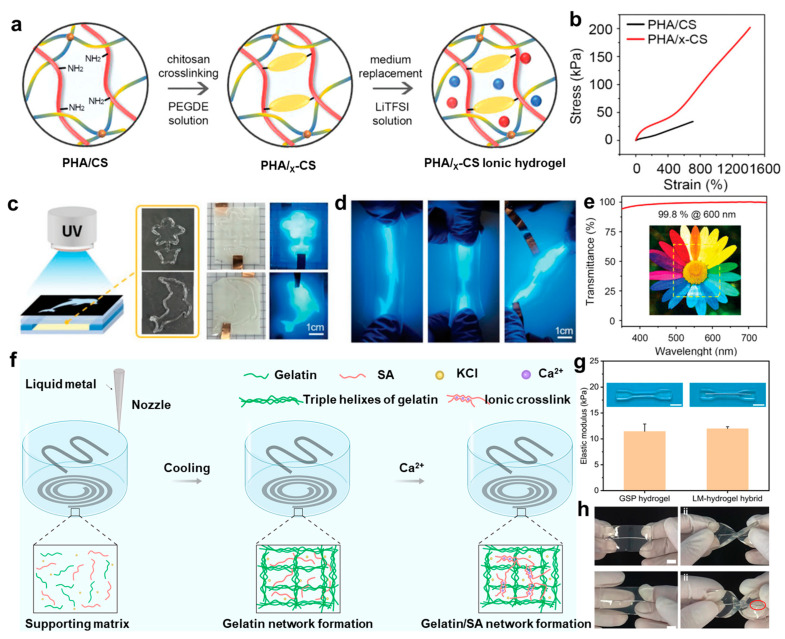
Fabrication strategies and structural characterization of chitosan-based hydrogels. (**a**) Schematic illustration of the fabrication process for PHA/x-CS hydrogels. (**b**) Representative tensile stress–strain curves comparing PHA/CS and PHA/x-CS hydrogels. (**c**) Schematic of the photomask-assisted patterning approach for PHA/x-CS hydrogels. (**d**) The mechanical deformation of the patterned hydrogel under stretching. (**e**) Optical transmittance spectrum of the PHA/x-CS hydrogel [42]. (**f**) Schematic of in situ 3D printing of LM-hydrogel hybrids. (**g**) Elastic modulus comparison between GSP hydrogel and LM-hydrogel hybrid. (**h**) Twist deformation comparison of LM–hydrogel and metal–hydrogel composites [34]. All pictures have been adopted with permission.

However, challenges remain in achieving precise control over the mechanical and electrical properties of chitosan-based hydrogels while maintaining their structural integrity under dynamic conditions. Go et al. [42] proposed a dual-network hydrogel system composed of poly(N-hydroxyethylacrylamide-co-acrylamide) and crosslinked chitosan (PHA/x-CS), fabricated via a lithium electrolyte-induced dual crosslinking mechanism (Figure 6a) to overcome this challenge. This system combines dynamic covalent bonding and ion–dipole interactions, enabling refined control over network topology. Compared with conventional single-network hydrogels, this dual-network structure significantly improves mechanical extensibility, shape flexibility at room temperature, and optical transparency (Figure 6b–e). Furthermore, the integration of photopolymerization-assisted shaping technology introduces a novel route for the personalized fabrication of flexible bioelectronic devices. An alternative approach was reported by Jiao et al. [34], in which an in situ 3D-printed gelatin–sodium alginate–potassium chloride (GSP)/liquid metal (LM) hybrid hydrogel system was developed. This strategy achieves in situ encapsulation of LM through a calcium ion diffusion-induced phase transition (Figure 6f), thereby eliminating the need for traditional multi-step transfer processes. Compared with conventional fabrication techniques, this method integrates the supporting matrix, conductive pathways, and biological interface functionalities into a single crosslinking step, offering the advantage of rapid curing. Therefore, the hydrogel exhibits mechanical compliance with biological tissues and maintains stable electrochemical interface characteristics (Figure 6g,h), thereby offering essential technological support for the advancement of next-generation fully flexible bioelectronic devices.

#### 2.2.2. Cellulose

Cellulose, a natural fiber derived from wood, cotton, hemp plants, agricultural waste, or bacterial synthesis, possesses a backbone composed of D-glucose units linked by *β*-1,4-glycosidic bonds [93,94]. Owing to its intrinsic biodegradability, water absorption capability, and biocompatibility, cellulose is widely employed to construct hydrogels through hydrogen bonding and physical or chemical crosslinking with other polymers, forming 3D networks [52,84]. In addition, molecular modifications, such as oxidative modification and graft copolymerization, have been utilized to enhance its functional versatility [95,96].

**Figure 7:**
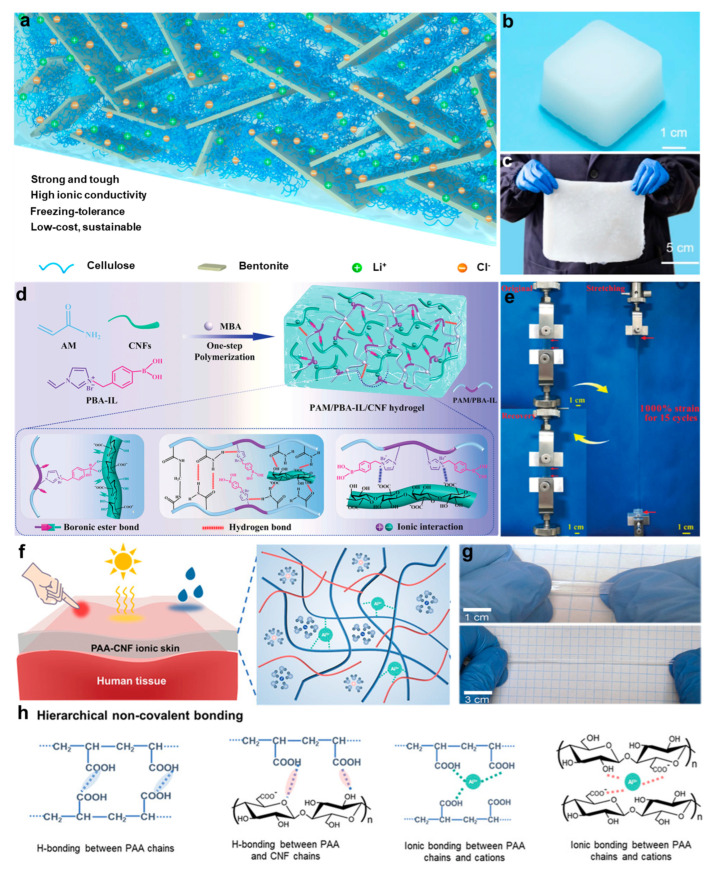
Preparation strategies and structural characterization of cellulose-based hydrogels. (**a**) Schematic of hierarchical microstructure in cellulose/BT hydrogels. (**b**) Photograph of the synthesized ion–CB hydrogel. (**c**) Optical image demonstrating the scalable fabrication of ion–CB hydrogels [99]. (**d**) Schematic of fabrication and dynamic network structure of PAM/PBA-IL/CNF hydrogels. (**e**) Tensile deformation of PAM/PBA-IL/CNF hydrogels under stress. (**f**) Design schematic of PAA-CNF-IL-H_2_O ionogels. (**g**) Visual of tensile performance of PAA-CNF-IL-H_2_O ionogels. (**h**) Molecular schematic of noncovalent bonding in PAA-CNF-IL-H_2_O ionogels [101]. All pictures have been adopted with permission.

However, balancing electrical conductivity and mechanical durability remains a key challenge in conventional cellulose-based hydrogels [97,98]. To address this challenge, Wang et al. [99] adopted a supramolecular engineering strategy to construct a cellulose/bentonite (BT) nanoconfined system. By forming multidentate coordination bonds between cellulose hydroxyl groups and aluminum oxide octahedra in BT, an intercalated structure was established (Figure 7a), which significantly overcomes the mechanical conductivity tradeoff. The resulting all-natural hydrogel’s (Figure 7b) anti-freezing capability and scalable processing features (Figure 7c) were validated in low-temperature wearable strain sensors, demonstrating substantially enhanced multifunctional performance through nanoscale structure control. In addition, utilizing phenylboronic acid–ionic liquid (PBA-IL) chemistry, Yao et al. [52] developed a dynamic semi-interpenetrating network hydrogel. A one-pot method enabled the uniform incorporation of cellulose nanofibrils (CNFs) into the PBA-IL/AM crosslinked matrix (Figure 7d). This system exploits dynamic boronate ester bonds along with electrostatic interactions and hydrogen bonding to achieve ultra-high stretchability (Figure 7e) and a self-healing efficiency of 92%, while maintaining excellent adhesion and optical transparency. Furthermore, Ye et al. [100] proposed a mechanical conductivity synergistic optimization strategy based on regulating hydration equilibrium within ionic liquid gels (Figure 7f). Through the competitive interactions between CNF and the IL/H_2_O system, the dissolution of CNF was effectively inhibited, while maintaining its nanoreinforcing effects. As a result, high-performance ionic gels exhibiting super-stretchability (Figure 7g), covalently crosslinked toughness (Figure 7h), and stable ionic conductivity were successfully developed. Compared with conventional hydrogels, these materials demonstrate substantially enhanced environmental stability under a wide temperature range and low-humidity conditions, thereby overcoming the common limitations of freezing-induced or dehydration-induced performance degradation.

#### 2.2.3. Alginate

Alginate is a naturally derived polysaccharide extracted from brown algae, featuring linear copolymer chains of *β*-D-mannuronic acid (M) and *α*-L-guluronic acid (G) residues joined by 1,4-glycosidic linkages [43,102]. The M/G ratio plays a pivotal role in determining the pore architecture and swelling capacity of alginate-based hydrogels, both of which are key factors for optimal bioelectronic functionality. These hydrogels can be rapidly fabricated through ionic crosslinking with divalent cations, such as calcium ions (Ca^2+^) [102,103]. However, its conventional single-network architecture results in limited mechanical strength and high brittleness, constraining its practical application [104,105]. To overcome these shortcomings, various strategies have been developed to improve the mechanical robustness and ionic conductivity of alginate-based hydrogels. Specifically, incorporating gelatin, polyvinyl alcohol (PVA), or carbon nanomaterials has enhanced mechanical strength and tuned ionic conductivity within the range of 10^−3^ to 10^1^ S/m [29,82,103,106].

**Figure 8:**
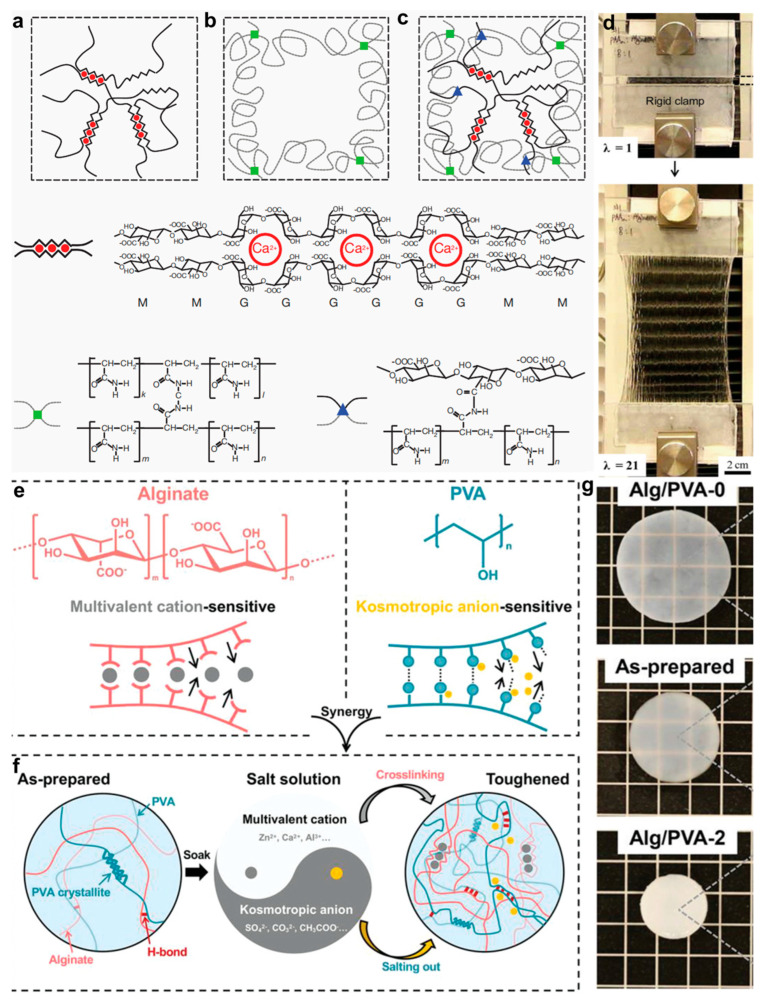
Structural mechanisms and enhanced mechanical performance of alginate-based hydrogels. (**a**) Schematic illustration of ionic crosslinking in Alg hydrogels. (**b**) Covalent crosslinking architecture in PAAm hydrogels formed via free-radical polymerization. (**c**) Hybrid Alg/PAAm network with ionic and covalent crosslinks. (**d**) Mechanical performance of the hybrid hydrogel [43]. (**e**) Schematic of dual crosslinking in Alg/PVA hydrogels. (**f**) Reinforcement strategy using ionic crosslinking and salting-out in Alg/PVA hydrogels. (**g**) Visual comparison of Alg/PVA hydrogels in different treatment states [106]. All pictures have been adopted with permission.

However, a substantial gap remains between the mechanical robustness of these hydrogels and that of native biological tissues, which restricts their broader clinical deployment. Suo et al. [43] addressed these limitations by designing an ion–covalent double-network hydrogel that overcomes the brittleness and poor extensibility of traditional systems (Figure 8a–d). This design integrates the crack-bridging effect of a covalent network with the dynamic dissociation–recombination behavior of ionic bonds, enabling hydrogels with 90% water content to achieve over 20 times the elongation at break and a rubber-like fracture energy of 9000 J/m^2^. The covalent framework provides shape-memory behavior, while the dynamic ionic network reduces notch sensitivity via energy dissipation, jointly contributing to superior mechanical performance. Additionally, Cui et al. [106] tackled the dual challenge of enhancing both conductivity and mechanical toughness in ionic hydrogels. They proposed a synergistic mechanism involving both anionic and cationic contributions. A physical double-network hydrogel composed of ion-sensitive polymers was immersed in a tailored salt solution, inducing dual crosslinking and salting-out effects (Figure 8e,f). This treatment dramatically improved the gel’s original fracture energy, ionic conductivity, and tensile strength by factors of 1100, 4.9, and 530, respectively. Consequently, a super-conductive hydrogel was achieved, exhibiting a tensile strength as high as 15 MPa, a toughness of 39 kJ/m^2^, and an ionic conductivity of 1.5 S/m. Therefore, this approach is broadly applicable across various salt-ion polymer systems and provides a fundamental breakthrough in overcoming the mechanical durability bottleneck of traditional conductive hydrogels, thus laying a foundation for wearable and implantable devices capable of operating under extreme mechanical conditions.

#### 2.2.4. Starch

Starch-based hydrogels, derived from natural starch, are polymeric 3D network structures formed through physical, chemical, or enzymatic modifications. Their physicochemical behavior is primarily governed by the ratio of amylose to amylopectin and the specific crosslinking techniques employed [107,108]. Due to the hydroxyl-rich nature of starch molecules, these hydrogels exhibit high hydrophilicity, biocompatibility, and biodegradability.

**Figure 9:**
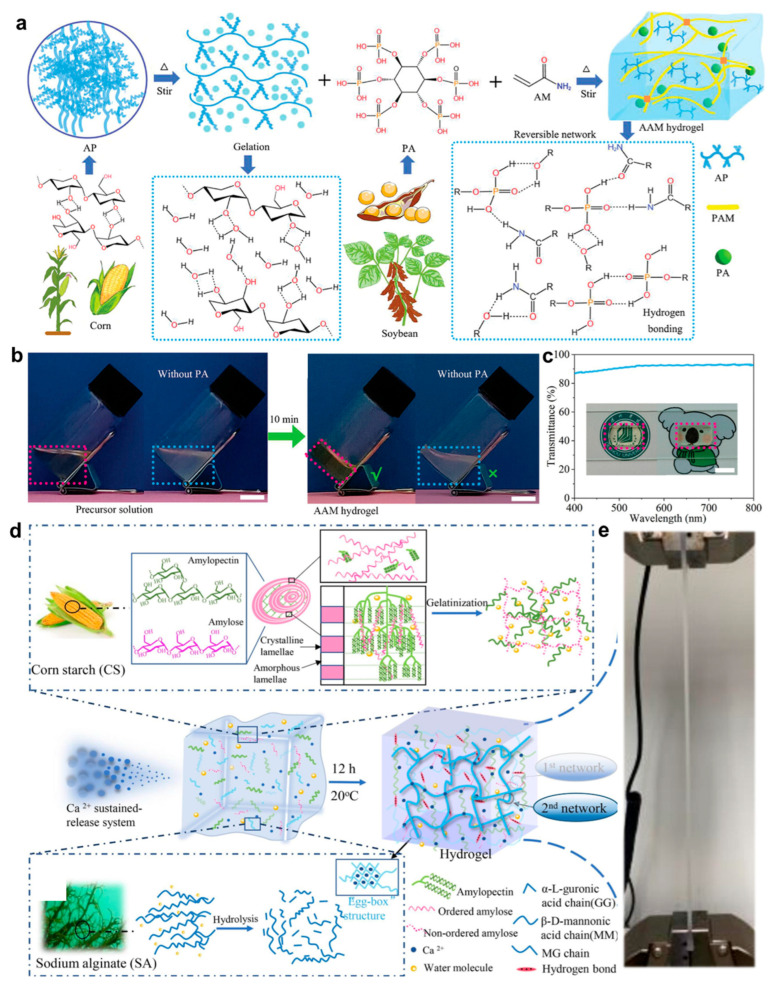
Preparation strategies and characterization of starch-based hydrogels. (**a**) Schematic of AAM hydrogel fabrication process. (**b**) Photographs of AAM hydrogels before and after thermal treatment. (**c**) Optical transmittance spectra of AAM hydrogels [114]. (**d**) Formation mechanism of CS-SA-Ca^2+^ hydrogel via ionic crosslinking. (**e**) Tensile deformation behavior of CS-SA-Ca^2+^ hydrogel [108]. All pictures have been adopted with permission.

However, their mechanical properties remain relatively weak [109,110,111]. To improve mechanical strength and self-healing capacity, a number of techniques have been used, including adding cellulose or polyvinyl alcohol and creating dynamic connections. Additionally, the incorporation of conductive materials such as polyaniline and MXene or the formation of ionic networks has been utilized to impart or enhance electrical conductivity [112,113]. To address the inherent limitations of traditional starch-based hydrogels, Zhao et al. [114] constructed a dynamic dual-mode crosslinked hydrogel composed of amylose and polyacrylamide, using a phytic acid-mediated hydrogen bonding strategy (Figure 9a). In this system, phytic acid molecules act as multidentate hydrogen bond donors, forming a reconfigurable physical network with polysaccharide chains, while trace amounts of covalent crosslinking are introduced to enhance structural stability. Here, the material achieves an elongation at break as high as 36,000% and an energy dissipation capacity of 47 MJ·m^−3^ while maintaining superior mechanical properties compared with conventional hydrogels. The dynamic hydrogen bond network is shown in Figure 9b. Figure 9c presents the material’s optical and environmental stability, including 93% light transmittance, freeze resistance at −20 °C, and seven-day moisture retention. In addition, Su et al. [108] proposed a green strategy for constructing a corn starch/Alg dual-network hydrogel through a calcium ion-induced synergistic toughening mechanism (Figure 9d). By combining the rigid segments of high-amylose starch with the flexible groups of Alg and applying gradient calcium ion crosslinking, a dynamic network structure was established via hydrogen bonding and ionic interactions. This design endows the hydrogel with room-temperature self-healing capability and rapid shape recovery driven by its porous structure, as shown in Figure 9e. Compared with conventional starch hydrogels, this system exhibits substantially enhanced mechanical robustness and dynamic responsiveness, offering promising potential for wearable and implantable bioelectronic applications. In order to facilitate a more intuitive understanding of the differences between different biomaterial-based hydrogels in terms of key performance parameters, this paper compiles a comparison of common hydrogels in terms of conductivity, Young’s modulus, and biocompatibility, as shown in Table 1.

## 3. Application

With their excellent biocompatibility, tissue-like mechanical compliance, and tunable electrical properties, protein and polysaccharide-based hydrogels have shown great promise for application in next-generation bioelectronic technologies. These materials enable intimate and stable interfaces with biological tissues, making them ideally suited for both wearable and implantable applications. In wearable bioelectronics, hydrogel-based devices can conform to the skin surface for non-invasive, continuous monitoring of physiological and biochemical signals, supporting personalized health management and early disease detection. In implantable bioelectronics, their biodegradability and biological functionality allow for seamless integration into internal tissues, enabling high-fidelity recording and targeted stimulation of biological activity. Recent advancements have propelled hydrogel-based systems into diverse biomedical applications, including electrophysiological signal monitoring and closed-loop therapeutic interventions, illustrating their transformative role in future bioelectronic technologies [122,123,124,125]. Although biomaterial-based hydrogels show promising applications in flexible electronics, they still face several key challenges. For example, their mechanical strength is typically lower than that of synthetic polymer hydrogels, limiting their stable use in high-stress environments, and furthermore, the poor physical stability of such hydrogels under humidity and temperature variations may affect their reliability in long-term wear or in vivo environments.

### 3.1. Wearable Bioelectronics

Recent advances in flexible and wearable bioelectronic devices have driven extensive research efforts toward their application in personalized medicine, HMI, energy harvesting, and health monitoring [126,127,128,129,130,131,132]. Due to their superior flexibility, tunable functionality, and biocompatibility, hydrogels derived from biological sources have garnered significant attention [133,134,135].

However, challenges remain in achieving reliable mechanical adaptation and stable electrochemical performance under dynamic bodily movements and complex physiological conditions [136,137]. To address these challenges, hydrogels derived from natural or synthetic biomaterials have been molecularly designed and engineered, enabling precise regulation of mechanical properties and electrochemical characteristics [138,139,140]. In addition, strategies such as network crosslinking, incorporation of conductive nanomaterials, and surface functionalization have been employed to substantially enhance the ionic conductivity and mechanical resilience, which are critical for bioelectronic interfaces [141,142]. Therefore, hydrogel systems offer a promising platform for the development of next-generation bioelectronic devices that operate reliably within complex biological environments. In this paper, we not only systematically sort out the core advances in current hydrogel research, but also systematically summarize the cutting-edge directions, such as bioelectronic fusion. Compared with the existing literature, we especially emphasize the potential of hydrogels in cross-cutting applications such as smart medicine and tissue interfaces.

#### 3.1.1. Electrophysiological Signal Monitoring

Physiological electrical signals, such as electrocardiogram (ECG), electroencephalogram (EEG), and electromyogram (EMG), are essential indicators of human physiological activities [143,144,145,146]. Accurate monitoring of these signals is critical for disease diagnosis, health management, and HMI [147,148]. However, conventional rigid electronic devices encounter substantial obstacles in dynamically interfacing with biological tissues, primarily due to mechanical mismatch, high interface impedance, and poor biocompatibility [149,150,151]. These limitations often result in signal attenuation and failure during long-term monitoring. Therefore, alternative materials that can form stable, tissue-adaptable interfaces are highly desired for reliable physiological signal acquisition [152,153,154,155]. In addition, dynamic crosslinking networks and ionic conductivity further enhance their potential for continuous bioelectronic monitoring.

**Figure 10:**
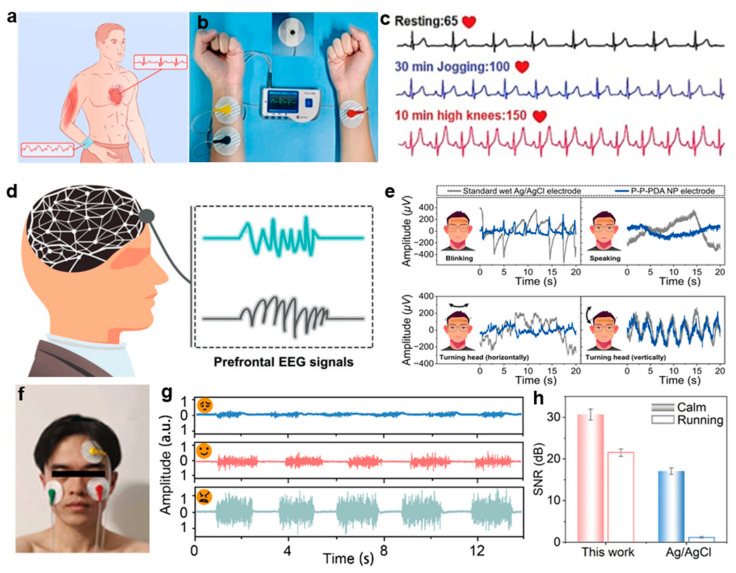
Applications of biomaterial-based hydrogels in physiological signal monitoring. (**a**) Schematic of PAC_2_V_3_ hydrogel electrodes for ECG monitoring. (**b**) Diagram of the ECG monitoring mechanism. (**c**) ECG signals from PAC_2_V_3_ electrodes under three physiological states [31]. (**d**) Structural layout of the EEG monitoring system employing hydrogel-based components. (**e**) Comparative analysis of motion artifacts between P-P-PDA nanoparticle electrodes and conventional wet Ag/AgCl electrodes [32]. (**f**) Setup for facial EMG recording with hydrogel electrodes. (**g**) EMG signals for different facial expressions. (**h**) Comparison of SNR of Ag/AgCl and hydrogel electrodes under different conditions [141]. All pictures have been adopted with permission.

Here, recent innovations in hydrogel-based sensing interfaces are highlighted through representative examples. For example, Zhu et al. [31] developed a cellulose nanofiber/polyionic liquid composite hydrogel (PAC_2_V_3_), which features an ordered honeycomb structure and dynamic ionic bonding synergy. This design enables high stretchability and low hysteresis, characteristics essential for wearable bioelectronics. The hydrogel demonstrated superior performance in dynamic ECG monitoring, accurately capturing heart rate fluctuations during resting, jogging, and jumping (Figure 10a–c). In addition, a wireless transmission system integrated with PAC_2_V_3_ facilitated real-time physiological signal interaction, providing an integrated interface solution for wearable health management applications. In parallel, Han et al. [32] introduced dopamine-modified polyvinylalcohol/polyvinylpyrrolidone (P-P-PDA) hydrogel electrodes, which were fabricated via an in situ nanoparticle formation strategy induced by peroxide. The resulting hydrogels exhibit a tissue-like modulus and stable adhesion interfaces, which are essential for long-term dynamic monitoring. As shown in Figure 10d, the multi-channel wireless system incorporating P-P-PDA hydrogel electrodes achieves superior anti-interference performance in EEG monitoring, effectively minimizing artifacts induced by blinks, speech, and head movements. Compared with commercial electrodes, the system exhibits a significantly lower signal fluctuation amplitude (Figure 10e), making it suitable for applications such as consciousness assessment and neurological diagnostics. Furthermore, inspired by the hierarchical structure of myelin, Liu et al. [141] designed PEDOT-modified sulfonated cellulose hydrogels, incorporating biomimetic conductive channel architectures. This approach imparts the material with exceptional stretchability, strong skin adhesion, self-healing ability, and low electrochemical impedance (Figure 10f–h). The hydrogel demonstrates stable, high-fidelity signal acquisition in facial EMG monitoring and outperformed commercial electrodes in ECG recording.

#### 3.1.2. Biochemical Signal Monitoring

**Figure 11:**
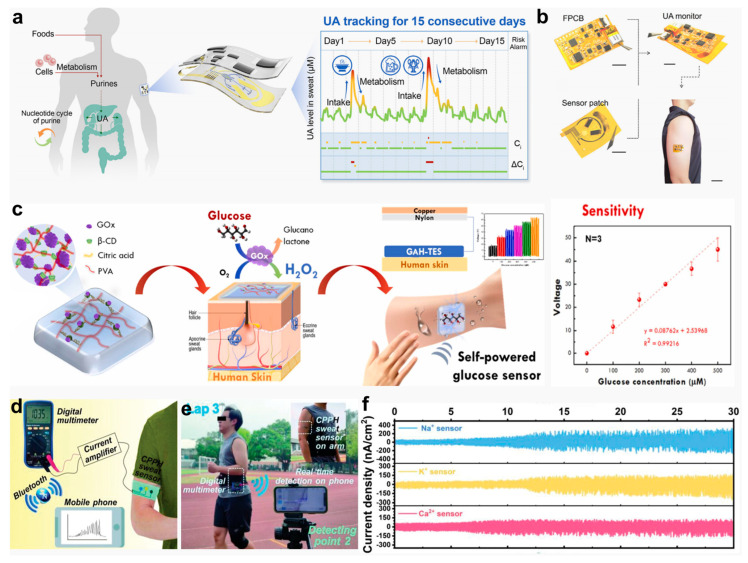
Hydrogel-based biochemical sensors and their physiological monitoring capabilities. (**a**) Schematic of a wearable device for UA monitoring. (**b**) Photographs of the UA sensor patch and its on-skin application [35]. (**c**) Diagram of a self-healing, glucose-adaptive hydrogel triboelectric sensor for sweat analysis [36]. (**d**) System connectivity of the CPPH sweat sensor with wireless components. (**e**) Real-time wireless sensing performance during running. (**f**) Ion detection profiles (Na^+^, K^+^, Ca^2+^) over 30 min of exercise [160]. All pictures have been adopted with permission.

In the field of bioelectronics, the monitoring of biochemical electrical signals serves as a critical bridge between physiological activities in the human body and the functional realization of electronic devices [135,142,156,157,158]. Hydrogel-based bioelectronic sensors, which can be functionally modified, enable continuous monitoring of target biomarkers such as neurotransmitters, lactate, and glucose [123,158,159]. Hu et al. [35] designed a hydrogel-based wearable biosensing platform based on hydrogels for long-term, non-invasive sweat uric acid (UA) monitoring. This platform utilizes a reusable sensing patch combined with a sensor design that mitigates antioxidant accumulation, effectively eliminating environmental interference and ensuring accurate and stable real-time detection (Figure 11a). The UA sensor is constructed on a flexible substrate featuring vertically stacked subcomponents (Figure 11b), minimizing skin contact to the greatest extent. Kanokpaka et al. [36] proposed a triboelectric sensor based on self-healing glucose-responsive hydrogels for continuous non-invasive glucose monitoring in sweat. The sensor encapsulates glucose oxidase (GOx) in *β*-cyclodextrin, embedded in a PVA matrix. The enzymatic conversion of glucose produces gluconic acid and hydrogen peroxide, elevating the local ionic strength, which induces the hydrogel’s swelling and the breaking of mechanochemical bonds, enhancing its conductivity. This conductivity change is converted into detectable signals by a triboelectric nanogenerator system, and as glucose concentration increases, the signal output performance improves (Figure 11c). Qin et al. [160] developed a self-healing, stretchable conductive TOCNF/PANI-PVAB hydrogel (CPPH) material based on dynamic crosslinking technology, fabricated using polyaniline with TEMPO-oxidized CNF and a PVA/borax composite. Following encapsulation in polydimethylsiloxane (PDMS), the hydrogel was coupled with ion-selective membranes to fabricate a fully flexible, self-powered sweat sensor capable of selectively detecting Na^+^, K^+^, and Ca^2+^ ions. The sensor system transmits electrical signals from the CPPH hydrogel through a current amplifier to a digital multimeter, which then relays data wirelessly via the smartphone’s Bluetooth module (Figure 11d). To evaluate system functionality, the device was worn by a subject while running, and real-time wireless monitoring of Na^+^ concentration in sweat was performed (Figure 11e). Figure 11f demonstrates the current signals of Na^+^, Ca^2+^, and K^+^ in sweat 30 min post-exercise, with signal intensity steadily increasing in tandem with the subject’s perspiration.

In addition to this, the application of hydrogels in wound healing sensors and diabetes detection systems has received much attention, demonstrating their great potential in flexible bioelectronics. Such sensors not only help to dynamically assess the state of wound healing, but also enable personalized interventions and adjustment of treatment strategies. In the field of diabetes management, hydrogels have been used to develop non-invasive or minimally invasive glucose detection platforms that enable continuous monitoring of glucose concentration in body fluids through integration with enzymes or photosensitive materials. Such systems are highly flexible and wearable, adapting to be attached to the skin surface for extended periods of time, providing diabetic patients with a more convenient and non-invasive means of blood glucose management.

### 3.2. Implantable Bioelectronics

Implantable bioelectronic devices are required to achieve long-term compatibility with biological tissues and stable transmission of electrophysiological signals [125,161]. This requirement imposes stringent demands on the materials used, particularly in terms of biocompatibility, interfacial matching, and controllable degradation behavior [162,163]. However, conventional electronic materials often suffer from mechanical mismatch and poor degradability, leading to adverse tissue responses and limited device lifespan [164,165]. To address these challenges, biopolymer-based hydrogels have been developed as promising materials for next-generation implantable devices. These materials mimic the extracellular matrix, offering tissue-like softness, tunable degradation profiles, and low immunogenicity, all of which are essential for seamless tissue integration. Through chemical modification and network design, parameters such as ionic conductivity, Young’s modulus, and degradation kinetics can be precisely controlled, enabling the fabrication of hydrogel systems tailored for specific biomedical applications [151,166].

Moreover, to achieve stable and flexible coupling between hydrogel systems and conventional electronic components, several interfacial engineering strategies have been developed. These include the incorporation of adhesion-promoting interlayers such as polydopamine and polyimide to strengthen bonding between hydrogels and inorganic surfaces, as well as the construction of dual-network architectures and ionic-conducting hybrid systems to reconcile mechanical compliance with electrical performance. For material applications, flexible conducting polymers such as PEDOT: PSS, liquid metal particle complexes, and surface-functionalized carbon nanomaterials have shown excellent interfacial adaptation and electrical coupling capabilities in several studies. Therefore, these hydrogels offer a substantial advancement over conventional materials in achieving superior biointegration and functional performance.

#### 3.2.1. Bioelectric Recording

Bioelectrical signal recording is a fundamental modality for bidirectional communication between biological systems and electronic devices, typically achieved through invasive or non-invasive electrode interfaces [167,168,169]. Among these, epidermal electrodes have emerged as the most widely utilized in clinical practice due to their non-invasive nature [44,170,171]. For example, large-area epidermal electrodes are routinely employed in EEG, which represents a mainstream method for monitoring cerebral activity. In addition, the acquisition of cardiac and muscular signals, such as ECG and EMG, respectively, also relies on epidermal electrodes, which have become standard tools in clinical diagnostics [124,172]. However, conventional epidermal electrodes are often limited by insufficient mechanical compliance and poor long-term stability at the bio-interface, particularly under dynamic physiological conditions.

**Figure 12:**
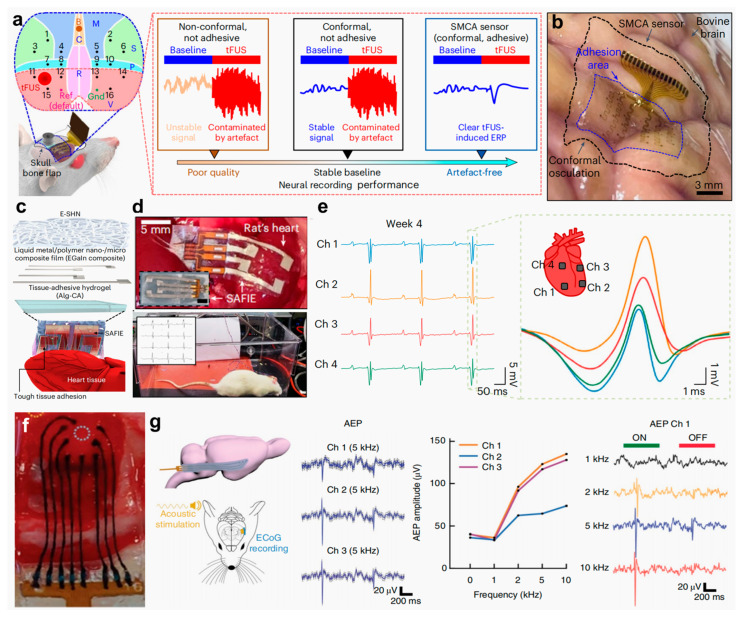
Biomaterial-based hydrogel electrodes for implantable electrophysiological recording. (**a**) Schematic of an in vivo material performance testing setup. (**b**) Images showing ultraconformal adhesion of the SMCA sensor on an ex vivo bovine cortex [15]. (**c**) Diagram of a SAFIE system on a rat heart. (**d**) Photographs of SAFIE deployed on cardiac tissue. (**e**) Real-time ECG recordings from four channels showing ventricular activity 4 weeks after implantation [37]. (**f**) Photograph of a viscoelastic array placed on rat cortical dura. (**g**) Schematic and data showing flexible array deployment and auditory cortex responses to sound stimulation [50]. All pictures have been adopted with permission.

To address these challenges, Lee et al. [15] proposed an innovative shape-adaptive cortical adhesive (SMCA) sensor for closed-loop ultrasonic neuromodulation in drug-resistant epilepsy. This system integrates a catechol-conjugated alginate (Alg-CA) hydrogel adhesive, a flexible 16-channel electrode array, and a self-healing polymer substrate, thereby enabling conformal adhesion to curved cortical surfaces and reliable neural signal acquisition in awake epileptic animal models during ultrasonic stimulation (Figure 12a). The SMCA sensor conforms intimately to irregular cortical geometries, forming topographic features of varying depth and demonstrating substantial potential as a brain–device interface (Figure 12b). In addition, Choi et al. [37] advanced this field by developing a strain-adaptive fiber-interlocked electronic (SAFIE) patch tailored for implantation applications. Central to this innovation is the synergistic design of a three-layered architecture (Figure 12c). A bottom layer composed of an Alg-CA ionic hydrogel provides tissue adhesion; a middle layer consisting of a gallium-indium LM/polymer nanocomposite ensures electrical conductivity, mechanical durability, and self-healing capacity; and a top layer fabricated via electrospinning forms a PDMS fiber-interlocked network, granting dynamic mechanical adaptability. Guided by an interfacial coupling strategy based on self-assembly, this patch conforms intimately to the cardiac surface, maintaining a stable electrical interface throughout prolonged operation (Figure 12d). Implantation studies revealed that the SAFIE patch could robustly capture ventricular depolarization delays over four weeks, with negligible deterioration in SNR (Figure 12e). Therefore, this decoupled yet integrated design of adhesion, conduction, and deformation offers a scalable architecture for organ-conformal bioelectronic systems. Additionally, Tringides et al. [50] further exemplifies the potential of hydrogel-based bioelectronics through the engineering of a flexible surface microelectrode array. In this design, traditional rigid encapsulation and metallic conductors are substituted with viscoelastic materials to achieve superior mechanical compliance with biological tissues such as the heart and cerebral cortex. The innovation lies in the use of carbon nanomaterial-enhanced Alg-CA hydrogels as ionically conductive paths, encapsulated within insulating hydrogel layers. Through precision fabrication processes, the resulting electrode arrays conform seamlessly to intricate organ topographies (Figure 12f) and remain fully compatible with conventional electrophysiological setups (Figure 12g). This approach presents a new paradigm in implantable electronics for both signal acquisition and stimulation.

#### 3.2.2. Bioelectrical Stimulation

Bioelectrical stimulation is widely employed as a versatile tool in both neuroscience research and clinical therapy [173,174]. Depending on the degree of invasiveness associated with tissue–electrode interactions, electrical stimulation modalities in biological systems are broadly categorized into invasive and non-invasive approaches. While implantable electrodes establish direct contact with target tissues, they typically entail higher levels of invasiveness. In contrast, epidermal bioelectronic stimulation techniques, such as transcutaneous electrical nerve stimulation, have been widely adopted for clinical pain management [33,54]. Despite the diversity in electrode configurations, the underlying physical principles governing electrical stimulation exhibit significant commonality across different modalities. However, conventional electrodes suffer from inherent limitations related to mechanical mismatch, limited biocompatibility, and unstable tissue–electrode interfaces.

**Figure 13:**
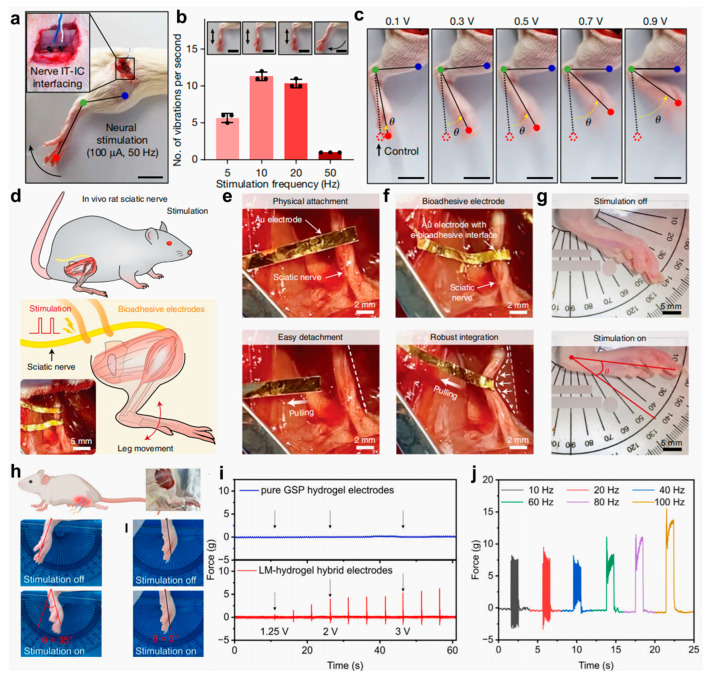
Schematic illustration and functional characterization of implantable electrical stimulation systems utilizing biomaterial hydrogel electrodes. (**a**) Photograph of neural stimulation using IT-IC hydrogel interfaces. (**b**) Correlation between leg vibration and stimulation frequency with motion snapshots. (**c**) Photographs showing ankle angle changes under different voltages [33]. (**d**) Schematic illustrations and an image of the sciatic nerve stimulation setup. (**e**) Physical attachment of a stimulation electrode on a sciatic nerve without the e-bioadhesive interface. (**f**) Robust integration of a stimulation electrode on a sciatic nerve with the e-bioadhesive interface. (**g**) Images of the ankle joint movement in response to electrical stimulation via the e-bioadhesive interface [172]. (**h**) Schematic of TA muscle stimulation using LM–hydrogel hybrid electrodes in rats. (**i**) Comparative images of ankle joint kinematics during TA muscle stimulation, employing LM–hydrogel electrodes versus pure GSP hydrogel controls. (**j**) Force output measurements under different TA muscle stimulation conditions [34]. All pictures have been adopted with permission.

To address these limitations, Jin et al. [33] designed an injectable conductive hydrogel (IT-IC hydrogel) utilizing a PB-mediated multi-crosslinking strategy. The hydrogel network was structurally reinforced through the synergistic combination of irreversible biphenyl crosslinks, gold nanoparticle (AuNP)-mediated coordination bonds, and multiple weak interactions, including hydrogen bonding and metal–π interactions. This composite conductive network substantially enhanced electrical signal transduction, facilitating not only intertissue conductivity but also seamless interfacing between tissues and bioelectronic devices. In addition, the hydrogel was integrated into a closed-loop robotic rehabilitation system to achieve precise neuromodulation and real-time motion feedback. As shown in Figure 13a–c, neural stimulation across a range of frequencies and amplitudes successfully elicited hindlimb muscle contractions without causing detectable tissue damage. Deng et al. [172] presented an innovative electrically conductive bioadhesive interface that enables rapid, strong, and reversible adhesion to wet and dynamically moving tissues while simultaneously maintaining electrical conductivity to facilitate bidirectional bioelectronic communication. The e-bioadhesive was evaluated in in vivo rat models, demonstrating excellent biocompatibility, as well as mechanical and electrical stability over time (Figure 13d–f). Notably, in vivo sciatic nerve stimulation using flexible electrodes combined with the bioadhesive interface produced consistent ankle joint motion, highlighting the interface’s potential to enhance tissue–device integration and improve the overall performance of implantable bioelectronic systems (Figure 13g). Furthermore, Jiao et al. [34] fabricated an LM–hydrogel composite that leverages the superior wettability and alloying properties of LMs, enabling seamless integration with conventional electronic interfaces while maintaining mechanical compliance (~10 kPa) consistent with soft tissues. This hybrid material supports the direct formation of functional electronic components without requiring additional encapsulation, thereby significantly advancing the application potential of hydrogels in bioelectronics. A schematic of the stimulation setup for the rat tibialis anterior (TA) muscle is shown in Figure 13h, and the corresponding muscle contraction forces under various stimulation intensities are presented in Figure 13i. The threshold voltage required to elicit TA muscle contraction using LM–hydrogel hybrid electrodes was as low as 1.25 V, compared with pure GSP hydrogel electrodes, which failed to generate measurable force even at 3 V. Figure 13j further details the muscle responses across different stimulation frequencies.

## 4. Conclusions and Future Perspectives

The integration of hydrogels derived from natural biomaterials into bioelectronics is regarded as a promising strategy for advancing next-generation healthcare technologies. In particular, polysaccharide and protein-based hydrogels are considered highly suitable for bioelectronic applications due to their inherent structural tunability and intrinsic biocompatibility. Through functionalization strategies such as conductive polymer blending, nanostructure engineering, and dynamic bond design, these hydrogels have been able to achieve critical performance metrics, including tissue-like mechanical compliance, efficient ionic and electronic conductivity, and strong interfacial adhesion. These features collectively allow seamless interfacing with biological tissues, which is essential for continuous health monitoring, precision diagnostics, and HMI. In wearable electronics and implantable sensors, such hydrogels have been demonstrated to bridge the gap between rigid electronics and soft tissues, thereby mitigating long-standing challenges related to mechanical mismatch and biological contamination.

However, key challenges persist in enhancing conductivity without compromising biocompatibility, ensuring long-term stability under physiological conditions, and achieving seamless integration with electronic components. Firstly, the reliance on conductive additives such as carbon nanotubes and conductive polymers to enhance conductivity has been found to compromise the mechanical integrity and biocompatibility of natural hydrogels. Therefore, future research should prioritize the exploitation of the intrinsic functionalities of proteins and polysaccharides to construct fully biomaterial-derived hydrogels with preserved conductivity and minimally impaired mechanical and biological performance. Secondly, although current hydrogel systems show promise in short-term applications, their long-term operational stability under dynamic physiological conditions, including enzymatic degradation and mechanical fatigue, remains insufficient for chronic implantation scenarios. In addition, challenges persist in the interfacial integration between hydrogels and electronic components, particularly in balancing electrical conductivity and biocompatibility, as well as ensuring stable signal transmission. Overall, the deep integration of natural biomaterial-based hydrogels with bioelectronic systems offers a unique pathway for the development of next-generation biocompatible medical devices. Owing to their structural tunability and inherent biocompatibility, polysaccharide- and protein-derived hydrogels have already demonstrated potential in flexible sensing platforms and soft-tissue interfaces. From a broader perspective, such materials are anticipated to reshape the landscape of medical electronics by converging with flexible machines and electronics, thereby driving forward the realization of personalized medicine and smart bioelectronic technologies.

## Figures and Tables

**Table 1:** Comparison of the properties of different biomaterial types of conductive hydrogels.

Hydrogel Materials	Conductivity (S m^−1^)	Young’s Modulus (kPa)	Biocompatibility	Refs.
Gelatin	1.83	30.4	Good	[26]
Gelatin	1.2	~100	Good	[115]
Collagen	~4	-	Good	[116]
Silk protein	0.244	22.2	Good	[117]
Gluten	~3.2	~60	Good	[79]
Chitosan	0.256	Skin-like	Good	[88]
Chitosan	1.93	950	-	[118]
Cellulose	3.4	900	Good	[119]
Alginate	0.22	1,290,000	Good	[120]
Alginate	~164.1	-	Excellent	[105]
Starch	1.47	8850	Good	[121]
Starch	0.1753	7090	Ideal	[110]

## Data Availability

Data sharing not applicable.
